# Authentication of the R06E Fruit Bat Cell Line

**DOI:** 10.3390/v4050889

**Published:** 2012-05-23

**Authors:** Ingo Jordan, Vincent J. Munster, Volker Sandig

**Affiliations:** 1 ProBioGen AG, Goethestr. 54, Berlin 13086, Germany; Email: volker.sandig@probiogen.de; 2 Rocky Mountain Laboratories, NIAID, NIH, 903 S 4th Street, Hamilton, MT 59840, USA; Email: vincent.munster@nih.gov

**Keywords:** fruit bat cell line, R06E, R05T, *Rousettus aegyptiacus*

## Abstract

Fruit bats and insectivorous bats are believed to provide a natural reservoir for a wide variety of infectious diseases. Several lines of evidence, including the successful isolation of infectious viruses, indicate that Marburg virus and Ravn virus have found a major reservoir in colonies of the Egyptian rousette (*Rousettus aegyptiacus*). To facilitate molecular studies on virus-reservoir host interactions and isolation of viruses from environmental samples, we established cell lines from primary cells of this animal. The cell lines were given to several laboratories until we realized that a contamination with Vero cells in one of the cultures had occurred. Here we describe a general diagnostic procedure for identification of cross-species contamination with the focus on Vero and *Rousettus* cell lines, and summarize newly discovered properties of the cell lines that may pertain to pathogen discovery.

## 1. Introduction

Infection with important zoonotic pathogens, including Nipah and Hendra viruses [1,2], Ebola, Marburg, and Ravn viruses [3,4,5], coronaviruses [6,7], and lyssaviruses [8,9,10] has been found in apparently healthy bats. Low pathogenicity may be due to coevolution of host and virus [11], and this may lead to a decrease in virus shedding, thus hampering isolation and propagation of live virus from environmental samples [12,13,14]. To facilitate epidemiological and biochemical studies of viruses adapted to fruit bats we immortalized primary cells from the Egyptian rousette (*Rousettus aegyptiacus*) and obtained the cell lines R06E, R05T and R05R [15]. Immortalization was achieved by transfection of the E1 region of human adenovirus 5. No artificial selection with antibiotics was applied at any time during the cell line generation process, and serial passaging confirmed complete immortalization.

New cell lines often stabilize with increasing passage number, and shifting properties of these lines were investigated at various points in the months after rescue of immortalized cell foci. Thereafter, R06E and R05T were provided to several laboratories until we realized that the supplied R06E cultures were not from Egyptian rousette but appeared to be Vero cells, suggesting either a contamination of the distributed R06E vials or an accidental switch of cell lines. During the investigation of this event we refined methods for detection of cross-species contamination and authentication of the fruit bat cell lines. We also observed unexpected properties in intentionally-spiked cultures, an ability for occluded contamination that we consider to be important due to the wide distribution of Vero cells, and present data suggesting that cell subpassage protocols and incubation temperature of infected cultures may be contributing parameters for successful isolation of viruses from field samples or recovery of recombinant viruses.

## 2. Results

### 2.1. Screen for Cross Species Contamination in Mammalian Cells

Contamination of cell lines is estimated to affect numerous cultures in research laboratories worldwide and the National Institutes of Health (NIH) has recently highlighted the importance of appropriate cell line authentication procedures for grant applications (notice NOT-OD-08-017; accessed March 2012 at http://grants.nih.gov/grants/guide/notice-files/NOT-OD-08-017.html). Although the authentication procedures are expected to vary with cell line, universal and robust approaches may at least facilitate initial screens. 

**Figure 1:**
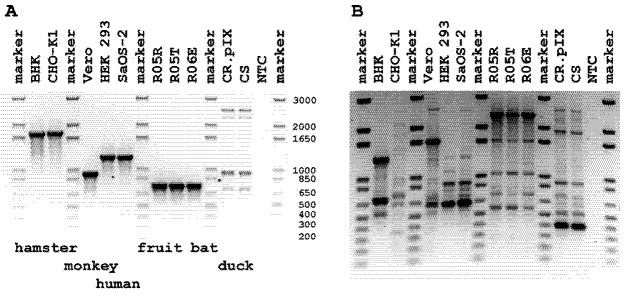
Species-specific PCRs. (**A**) Genomic DNA was isolated from different cell lines and analyzed by PCR using a primer pair designed to amplify across an intron of the conserved MACF1 gene; (**B**) Isolated genomic DNA was analyzed by a single-primer PCR to amplify minisatellites. Abbreviations for cell lines: BHK, Syrian baby hamster kidney; CHO-K1, Chinese hamster ovary; HEK 293, human embryonic kidney; SaOS-2, human osteosarcoma; R05R, R05T, R06E, *Rousettus aegyptiacus*; CR.pIX, CS, *Cairina moschata* retina or somites; Vero, African green monkey. NTC for non template control.

We tested several primer sets and found that PCR against minisatellites with primer YNZ22 [16] and within the MACF1 gene [17] are especially suitable for discriminating different cell lines (Figure 1). PCR fragment patterns are complex after amplification of minisatellites by a YNZ22 primer. This complexity allows the clear identification of cell lines and the ability to distinguish related cultures once reliable fingerprints have been established. The disadvantages are that contamination could be masked by the greater number of signals and that amplification conditions may influence the reliability of the obtained pattern [18]. To detect contamination in previously untested cell lines, changes in patterns have to be monitored over several passages, with the caveat that if original and contaminating cell lines are in equilibrium, a shift to one cell line may not be observed.

Microtubule-actin crosslinking factor 1 (MACF1) is important for the organization of the cytoskeleton and highly conserved among vertebrates. The structure of the gene is complex with a large number of exons [19]. The selected amplicon spans adjacent exons to yield a single band where size allows differentiation of members of different species. Although this signal can only contribute to species assignment it should be valuable in pointing to the potential presence of contaminating cells without prior knowledge about the original cell line.

### 2.2. Authentication of R06E

**Figure 2:**
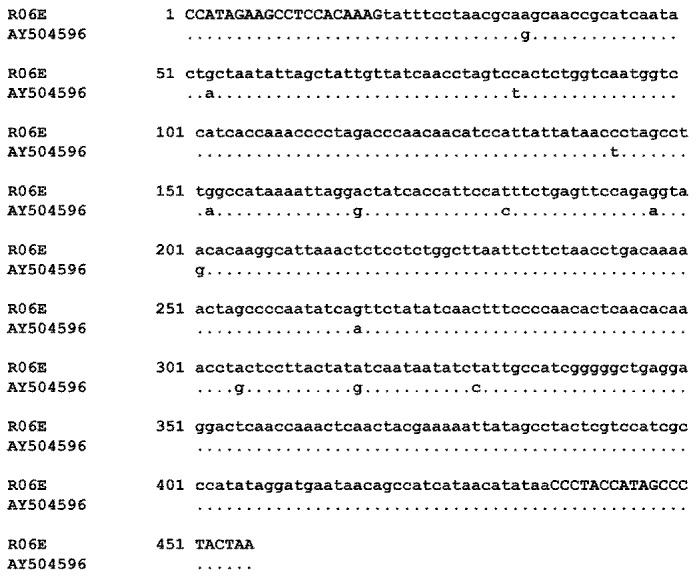
Comparison of ND2 sequences of R06E and GenBank AY504596 from the Egyptian rousette. The closest significant alignment is shown after query through NCBI BLAST (consensus sequence of three independent clones). All differences in nucleotide sequence to the GenBank entry are also present in the R05R and R05T sister cell lines. Capital letters: primer sequences.

Although a combination of PCR reactions can confirm identity, only sequencing can provide a reliable species assignment for previously uncharacterized cell lines. We therefore designed primers using the published sequence of the gene for the mitochondrial NADH dehydrogenase subunit 2 (ND2) from the Egyptian rousette (GeneBank accession number AY504596), and with these primers amplified and sequenced a gene fragment to confirm that R06E was isolated from such an animal (Figure 2).

### 2.3. Cocultivation of Vero.GFP and R06E Cells

At the time we investigated the tainted culture of R06E with the MACF1 primers we could identify only Vero cells and initially assumed that cultures must have been switched prior to cell banking. When we retested earlier cryovials the culture was pure, from fruit bat origin and apparently remained so for at least 12 passages. However, at passage 14 a faint signal for Vero cells was detected, becoming the dominant signal at passage 19, and the only signal with passage 23.

Vero and R06E cells proliferate with similar doubling times of 18 hours and to similar cell densities in the culture flasks. Following our surprise that under seemingly equal settings one initially non-detectable cell line completely displaces the other we generated Vero cells that stably express GFP to allow quantification of the process at a cellular level.

**Figure 3:**
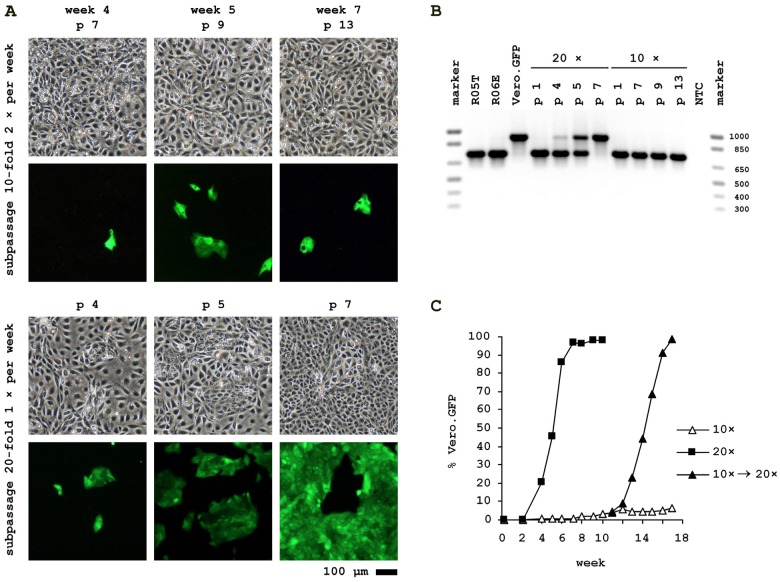
Cocultivation of Vero.GFP with R06E. The cocultures shown differ only by frequency of splitting. (**A**) Bright field and fluorescence pictures were taken at different passages after spiking of Vero cells, stably expressing GFP, into R06E cell culture; (**B**) Species-specific MACF1 PCR of fruit bat (R05T, R06E), monkey (Vero.GFP) and spiked cell cultures with different splitting procedures (as shown in A) at various passage levels; (**C**) Ratio of GFP-positive Vero to R06E in different cell cultures. Cells were quantified by FACS analysis.

Parallel cultures of 2.5 × 10^5^ R06E cells were spiked with approximately 50 of the Vero.GFP cells to simulate a low level contamination event. Antibiotic selection for transgene maintenance in Vero.GFP was discontinued 2 passages prior to cocultivation. One culture was subpassaged twice weekly by 10-fold dilution and the other culture once weekly by 20-fold dilution. Both protocols reflect common cell culture maintenance procedures. To track relative Vero.GFP content with each passage, fluorescence images were taken, FACS performed (at lower passages GFP-positive cells were counted by eye) and genomic DNA isolated for PCR against MACF1. As shown in Figure 3, the ratio of GFP-positive to -negative cells remained at a constant low level if cells are passaged twice a week. Detection of Vero.GFP cells with MACF1-PCR in this cell culture was not possible. However, when passaged only once a week Vero.GFP cells had already overwhelmed R06E cells after 7 passages of cell culture. If the culture with long-established, low-level contamination at week 10 is switched to the longer split intervals, Vero.GFP again increases rapidly from 3% to 98%. 

Control experiments performed but not shown: Vero.GFP spiked into and cocultivated with parental Vero cells eventually is lost independent of splitting procedures; the mean percentage GFP content measured throughout the experiment in a parallel Vero.GFP culture without antibiotic selection is 97.2%, and background levels in non-spiked parental R06E is 0.1%.

### 2.4. Variable Permissivity of R06E Cells

We reported previously that different *Rousettus* cell lines (R05T and R06E) are fully permissive for hyperattenuated poxvirus strain MVA (modified vaccinia Ankara), and that R06E at higher passage levels and opposed to the related R05T cell line has significantly reduced permissivity for MVA [15]. This observation is surprising, and although analysis of intermediate cryocultures indicated that the experiments were performed with pure R06E, we decided to confirm and expand on this result of the study.

**Figure 4:**
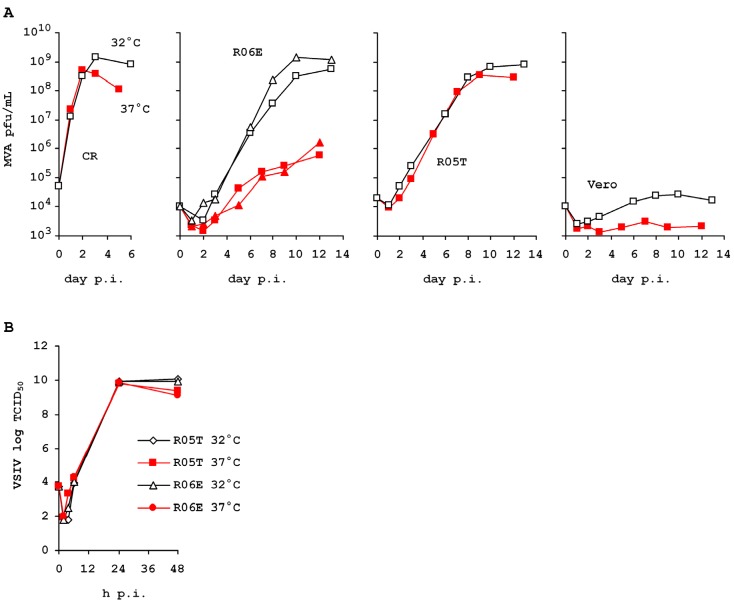
R06E at passages 50 and 90 has reduced permissivity for MVA (**A**) but not for VSIV (**B**) at 37 °C. R06E examined for VSIV was at passage 60. Cultivation and infection at 37 °C is highlighted in red with bold symbols, at 32 °C in black with open symbols. Lower passage R06E in the second panel of (**A**) is shown with squares and higher passage R06E is shown with triangles.

As shown in Figure 4, susceptibility of authenticated R06E to MVA is indeed very low, compared to R05T. However, virus replication and yield of infectious virus increased to expected levels at lower cultivation temperature. This property appears to have stabilized in the R06E cell line and does not change after further 40 passages. No such effect can be observed in the R05T.

Temperature attenuation has not been reported for MVA previously. This virus strain is derived from a wild type vaccinia virus that has been adapted to replication in avian cells and has lost replication potential in most mammalian cells [20,21]. Vero cells were therefore included as a negative control, and avian AGE1.CR [22] as a positive control within our experiment. In both cell lines MVA replicates as expected and viral propagation showed no obvious differences at both temperatures.

Permissivity for a virus in R06E is not generally restricted at conventional cultivation temperature but appears to be confined to a biochemical pathway of host and virus interaction not utilized by all viruses. As an example, vesicular stomatitis Indiana virus (VSIV) replicates to similar titers at both temperatures (Figure 4).

## 3. Experimental Section

### 3.1. PCR and Primers

DNA was isolated from 4 × 10^6^ cells with QIAmp DNA Blood Mini columns (Qiagen) according to the manufacturer's instructions. PCRs were performed with 100 ng of template DNA and 2 µM of primer. Cycle parameters were 35 repeats of 55 °C annealing for 30 s, 72 °C extension for 180 s and 94 °C melting for 20 s. MACF1 primer sequences are CCATCTgCTgAgTATAAAgTggTgAA and gCCTCCTTCTgCTTgAAgCA, single-primer PCR for amplification of minisatellites was performed with CTCTgggTgTCgTgC. MACF1 primers bind to human exons 61 and 62, but only the forward primer partially recognizes exon 54 in chicken (GenBank NC_006110). A reverse primer optimized for binding to exon 55 in chicken (ATCTAggAgCCgCTggAgCA) yields a single MACF1 band for duck at 0.9 kb but multiple bands on most mammalian genomic DNA. The studies described here were performed with the mammalian MACF1 primers. The duck cell lines were also authenticated with their respective ND2 sequence.

The ND2 fragment was amplified with primers CCATAgAAgCCTCCACAAAg and TTAgTAgggCTATggTAggg with 36 cycles of 56 °C annealing for 30 s, 70 °C extension for 60 s and 95 °C melting for 20 s with proofreading KOD polymerase (Takara Shuzo). The amplified fragment was cloned into pTOPO-Blunt (Invitrogen) and three independent clones were sequenced with M13 primers from both directions.

### 3.2. Cocultivation

All cells were maintained in DMEM:F12 (Invitrogen) supplemented with 5% fetal calf serum. Vero cells stably expressing GFP were created by transfection of PciI-linearized derivative of pEGFP-N1 plasmid using Effectene (Qiagen) and selection with 300 µg/mL G418, until clones were recoverable, and with 1000 µg/mL G418 thereafter. Antibiotic selection for transgene maintenance in Vero.GFP was discontinued 2 passages prior to cocultivation. Approximately 50 Vero.GFP cells were transferred into cultures of 2.5 × 10^5^ R06E cells. Both cultures were passaged in parallel, the culture in the upper panel of figure 3 split 10-fold twice weekly, the other 20-fold only once weekly. Ratio of GFP-positive cells were quantified by direct cell counting in the monolayer for the first few passages and by FACS with the CyFlow Space (Partec) unit at later stages. To perform FACS, cells were dislodged with TrypLE (Invitrogen), collected by centrifugation with 100 × g, washed once with PBS and finally resuspended in PBS. 50,000 cells were counted with instrumental gain set to 130 units for forward and side scatter, and to 165 units for the 488 nm excitation and 515–560 nm band pass channel measuring GFP. Flow rate was adjusted to 1000 counts per second and a gate based on forward and side scatter was set to exclude debris. Pure R06E and Vero.GFP without antibiotic selection were cultivated and assayed for GFP expression in parallel to the mixed culture.

### 3.3. Virus Infections

Cells were seeded 24 h prior to infection in 6-well plates, the smaller CR cells at 5 × 10^5^ cells per well, Vero, R06E and R05T at 1 × 10^5^ cells per well. Modified vaccinia Ankara, MVA (ATCC #VR-1508) was added directly to cell monolayers to a MOI of 0.1 and vesicular stomatitis Indiana virus, VSIV (FLI #RVB-030), to a MOI of 0.01. Seed virus titers were 1.2 × 10^8^ pfu/mL and 8.5 log tissue culture infectious doses (TCID_50_), respectively. Progeny virus was isolated by three cycles of freeze/thawing of full cell lysates. MVA was titered by focus immunostaining on Vero, as described previously [22]. VSIV was quantified by determining TCID_50_ values in CR cells, calculated from results of 6 repeats per sample using the Spearman and Kärber formula.

## 4. Discussion and Conclusions

Virus tropism can be determined by several host factors, such as availability of receptors and activation of cellular pathways important for viral gene expression or morphogenesis. For this reason some viruses are more efficiently isolated and studied if cell lines from cognate hosts or tissues are available [23,24]. Aiming to provide a new cell line hopefully permissive for a broad spectrum of viruses, and to provide a new substrate for isolation of pathogens with narrow host range, we immortalized cells derived from the fruit bat [15] that appears to be the natural reservoir for Marburg and Ravn viruses, the Egyptian rousette [5].

These cell lines may facilitate studies on species-specific molecular interactions of viruses associated with fruit bats as adaptation to non-cognate cell cultures sometimes induces unpredictable changes in viral particles or genomes [25,26] that possibly will not occur in reservoir-derived host cells. Conversely, certain cellular properties are easier to investigate if appropriate substrates and references are available. For example, innate immunity may modulate severity of disease and differences sometimes have been discovered even in closely related species (as has been shown for influenza A virus infection in chicken compared to duck, or Pekin duck compared to Muscovy duck [27,28]).

If pathogens that have coevolved with bats are to be studied in bat cell lines, the identity of the substrates to be used in the investigation is important. Yet, misidentification and cross-contamination of cell lines or deposited samples is estimated to affect numerous cultures in research laboratories worldwide [29,30,31,32,33]. The NIH has recently highlighted the importance of performing cell line authentication, especially if such cultures are essential to the work described in grant applications (notice number NOT-OD-08-017 and open letter referenced therein). Affected by cell line contamination in a R06E cell culture that we generated and distributed to others [34], we investigated cross species contamination to avoid any repeat of such a devastating problem in the future.

As shown in Figure 1 and Figure 3, the cross-species PCR with MACF1 gene-specific primers is a focused and reliable tool to confirm purity of secondary cultures and cell lines. The sensitivity of the method described here suggests that contamination can remain undetected until levels of 10–20% are reached. A similar threshold value is reported for isoenzyme analysis [35]. The PCR fragment pattern is more complex after amplification of minisatellites using the YNZ22 primer and thus suitable to contribute to genetic fingerprints of cell lines. The disadvantages are that contamination could be masked by the greater number of bands unless more precise methods such as capillary electrophoresis is applied to resolve the pattern, and that only cell lines with established genetic fingerprints can be authenticated. A conventional PCR with primers against MACF1 should point to interspecies contamination in mammalian cell cultures even if expected PCR fragment patterns are not known as any double band alerts to a potential problem. With respect to further authentication of our *Rousettus* cell lines, sequence of ND2 and suitable primers are shown in Figure 2.

Surprisingly, as visible in Figure 3, a contaminating cell line may either displace the original cell line or remain undetectable for many passages with conventional split protocols as the only determining parameter. Also, for this reason, repeated testing of cultures may be essential to confirm continued integrity of cell lines, again highlighting a need for simple procedures that can be applied repeatedly as opposed to the more complex and expensive genetic fingerprints.

The contaminant in a distributed R06E cell culture were Vero cells, a cell line that is widely used in many laboratories, and often in parallel to other cell lines as a reference, because of its susceptibility for a wide spectrum of viruses and deficiency in interferon signaling [36]. The potential for insidious contamination by Vero is unexpected and deserves attention. R06E is a fully established cell line and there appears to be no visible advantage for Vero.GPF cultures as lag phase and maximum cell densities are similar for the two cell lines, with replication even slightly faster for R06E. Vero cells derived from the World Health Organization 87-10 Cell Seed Bank have been tested for tumorigenicity and adventitious agents at low passage levels and are considered to be safe for vaccine production. However, capacity for cell migration and invasive phenotype has been described for this spontaneously immortalized cell line to increase with generation number [37], and the Vero cultures tested here are at high passage levels. For this reason, we suspect that very subtle differences in contact inhibition may allow Vero.GFP to continue cell division and accumulate where R06E enters cell cycle arrest. Such effects could explain why less frequent split rates with higher final cell densities favor Vero.GFP.

These results could be insightful also for studies of less obviously heterogenous populations. For example, recombinant viruses or agents in environmental samples may encounter a barely permissive cell culture until they have been adapted by serial passages [38,39]. The few infected cells harboring an unapparent virus could be seen analogous to Vero.GFP in our experiments. Appropriate variations in splitting procedures during blind passaging may support proliferation of positive cells and thereby eventual recovery of a virus that otherwise may remain undetected. A potential molecular mechanism for such a model could be found at critical nodes of crosstalk between cell cycle (modulated by splitting procedure or viral factors [40]) and innate immunity. One such node is occupied by the NF-κB family of transcription factors: activity of NF-κB changes with phase of the cell cycle [41,42] and at the same time this factor from within innate immunity and inflammation cascades interferes with [43] or augments [44] replication of certain viruses, and may even modulate latency [45].

Another aspect relates to phenotype variations within a cell culture. As an example, for Vero cells it has been described that splitting procedures influence emergence of tumorigenicity [46]. This observation may be related to the results summarized here if the population is heterogenous at onset, and if different phenotypes, such as low or absent tumorigenicity, react differently to initial or final cell densities. Such a potential heterogeneity has been described for another cell line important in vaccine research, the Madin-Darby canine kidney cells (MDCK) [47]. Mixed phenotypes are probably without consequences in most experiments, but may confound some interpretation if results of distant relatives of the same cell line are compared across laboratories.

We reported previously that R06E cells at higher passage levels and opposed to the related R05T cells appear to lose permissivity for hyperattenuated poxvirus strain MVA. These results are confirmed but another unexpected property of R06E cells compared to the R05T was observed: permissivity of R06E cells for MVA increased to expected levels at lower cultivation temperature. Vero cells remained non-permissive for MVA at low temperature, and the rhabdovirus VSIV replicated efficiently in R06E at normal and low cultivation temperatures. This specific example of virus-host interaction is unexpected and suggests that for adaptation or rescue of viruses from environmental samples, lower incubation temperatures should be included in the experimental variations to increase efficiencies.
